# Does the Addition of Strength Training to a High-Intensity Interval Training Program Benefit More the Patients with Chronic Heart Failure

**DOI:** 10.31083/j.rcm2401029

**Published:** 2023-01-16

**Authors:** Manal Alshamari, Christos Kourek, Despina Sanoudou, Dimitrios Delis, Stavros Dimopoulos, Nikoletta Rovina, Serafim Nanas, Eleftherios Karatzanos, Anastassios Philippou

**Affiliations:** ^1^Clinical Ergospirometry, Exercise & Rehabilitation Laboratory, 1st Critical Care Medicine Department, Evangelismos Hospital, School of Medicine National and Kapodistrian University of Athens, 10676 Athens, Greece; ^2^Department of Cardiac Rehabilitation, Heart Hospital, Hamad Medical Corporation, 3050 Doha, Qatar; ^3^Department of Cardiology, 417 Army Share Fund Hospital of Athens (NIMTS), 11521 Athens, Greece; ^4^Clinical Genomics and Pharmacogenomics Unit, 4th Department of Internal Medicine, School of Medicine, National and Kapodistrian University of Athens, 11527 Athens, Greece; ^5^Cardiac Surgery Intensive Care Unit, Onassis Cardiac Surgery Center, 17674 Athens, Greece; ^6^Department of Respiratory Medicine, National and Kapodistrian University of Athens, 11527 Athens, Greece; ^7^Department of Physiology, School of Medicine, National and Kapodistrian University of Athens, 11527 Athens, Greece

**Keywords:** high-intensity interval training (HIIT), strength training, cardiac rehabilitation, functional capacity, chronic heart failure (CHF), quality of life

## Abstract

**Background:**

Aerobic exercise, either continuous or high intensity interval training (HIIT), induces important benefits in chronic heart failure (CHF) patients. Resistance training has been also shown to be beneficial in CHF. However, data regarding combined aerobic exercise and muscle strength training is still limited. The aim of this study was to investigate whether adding strength training to a HIIT protocol within a cardiac rehabilitation (CR) program has a cumulative beneficial effect on the functional capacity (FC) and quality of life (QoL) in patients with CHF.

**Methods:**

Forty-four consecutive patients [35 males, ejection fraction (EF) <50%] with CHF under medication enrolled in a 36-session CR program and were randomized in two exercise groups; HIIT (HIIT group) or HIIT combined with strength training (high intensity interval training combined with strength training (COM) group). All patients underwent baseline and endpoint outcome measures of a symptom-limited maximal cardiopulmonary exercise testing (CPET), 1 repetition maximum (1RM) test, muscular endurance test, echocardiography, and Minnesota Living with Heart Failure Questionnaire (MLWHFQ).

**Results:**

Most of the CPET indices, EF, 1RM test, muscular endurance and QoL were improved after the CR program in each exercise training group (*p *< 0.05). However, COM group demonstrated a further improvement in chest muscle testing and workload at anaerobic threshold (AT) compared to HIIT group.

**Conclusions:**

An exercise-based CR program, consisted of either HIIT or HIIT combined with strength training, improves FC and QoL of patients with CHF. However, the addition of strength training to HIIT seems to have further beneficial effects on chest muscle strength and endurance, as well as workload at AT.

**Clinical Trial Registration:**

The study was registered in ClinicalTrials.gov with number NCT02387411.

## 1. Introduction

Chronic heart failure (CHF) is a clinical syndrome that remains the leading cause of mortality and morbidity worldwide [1, 2]. Its prevalence, according to the 2021 American Heart Association Statistical Update, is estimated at approximately 1.8% of the total US population and between 1% and 2% in Europe [3, 4]. CHF is characterized by impaired microcirculation [5, 6, 7, 8], vascular endothelial dysfunction [5, 6, 7, 8], exercise intolerance, reduced exercise capacity [9, 10, 11, 12], and reduced skeletal muscle mass [13, 14, 15, 16]. The effect and symptoms of CHF on individuals’ everyday routines are reflected in their health-related quality of life (QoL), which is usually decreased in these patients [17, 18].

During the last decades, studies investigating aerobic exercise are shown to improve microcirculation [19, 20] and vascular endothelial function [21, 22, 23], exercise capacity [21, 22, 23, 24, 25, 26], skeletal myopathy [19, 27, 28], and QoL [25, 29, 30] in CHF patients. The most recent 2021 European guidelines for managing and treating chronic heart failure, include a class IA recommendation for patients with CHF to engage in regular aerobic exercise protocols which are the most studied aspects of cardiac rehabilitation (CR) programs [31].

Data in the last two decades suggests high intensity interval training (HIIT) to induce at least comparable benefits to continuous regimes and also provides evidence on the benefits of combined aerobic and resistance exercise training protocols in CHF patients [32, 33, 34, 35, 36, 37, 38]. Combined regimes have been shown to induce additional benefits in terms of strength and aerobic variables [28, 39, 40, 41, 42], and therefore, resistance/strength training has been established as core component of cardiac rehabilitation in chronic heart failure [43, 44, 45, 46]. However, extending previous findings regarding the combination of HIIT and resistance regimes in heart failure, and adding new knowledge in literature, would be useful for the establishment of individualized exercise training programs.

We hypothesized that the addition of strength training to HIIT may provide further improvement in strength and exercise capacity in CHF patients. Thus, the aim of this study was to investigate whether adding strength training to a HIIT protocol has a cumulative beneficial effect on the functional capacity (FC) and muscle function indices, as well as on the QoL, in patients with CHF undergoing a CR program.

## 2. Materials and Methods

### 2.1 Participants

**Fig. 1.**
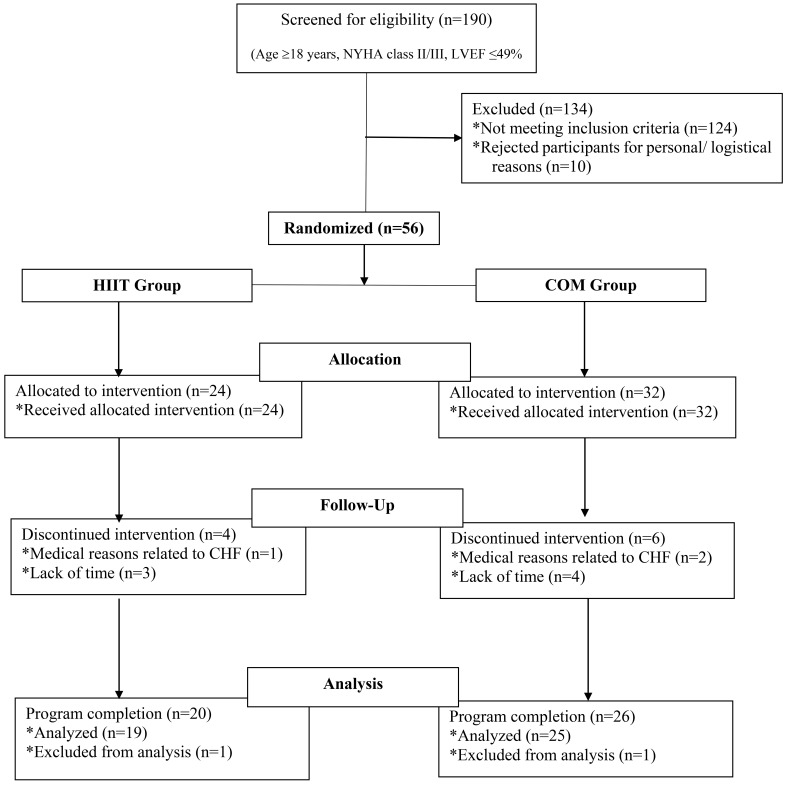
**Flow chart describing the process of the randomized clinical trial**. LVEF, left ventricular ejection fraction; NYHA, New York Heart Association; HIIT, high-intensity interval training; COM, combined training; Follow up, the period during the 36-session exercise training program.

Forty-four consecutive patients (35 males) with stable CHF under medication were recruited in the CR program. Inclusion criteria were: (i) New York Heart Association (NYHA) class II/III, (ii) age ≥18 years and (iii) a mildly-reduced or reduced ejection fraction (EF) ≤49%. Patients who met the compliance criteria and attended all exercise training sessions were included in the analysis. Exclusion criteria included: (i) severe valvulopathy, (ii) moderate or severe chronic obstructive pulmonary disease(COPD), (iii) severe peripheral angiopathy, (iv) neuromuscular diseases, and (v) any contraindications in performing a symptom-limited maximal cardiopulmonary exercise testing (CPET) (Fig. 1).

### 2.2 Study Design

This single-blinded, clinical randomized control study was performed between September 2015 and August 2019. It was approved by the Ethics Committee and the Administration Board of the hospital and it was in accordance with the Declaration of Helsinki. The study was registered in ClinicalTrials.gov with number NCT02387411. The CR program was a 3-month program consisting of 36 exercise training sessions, which were held three times a week. All patients from both groups performed 36 sessions in order to be included in the analysis.

All participants were asked to sign an informed consent. Patients were referred by outpatient HF departments of the biggest hospitals of the city. To determine their suitability for the program, including safety and feasibility of exercise, expert cardiologists evaluated their medical history, as well as laboratory and imaging exams, and performed clinical examination of the patients. Each patient underwent a symptom-limited maximal CPET on an electromagnetically braked cycle ergometer (Ergoline 800; SensorMedics Corporation, Anaheim, CA, USA) twice; before and after the CR program.

### 2.3 Exercise Capacity Assessment—Cardiopulmonary Exercise Testing

Individual work rate increments were calculated for an 8–12-minute test duration [47] at baseline and at the end of the rehabilitation program in each patient. During CPET, patients could breathe using a special mask with a low-resistance valve and an established gas mixture. While breathing, various breathing parameters including oxygen uptake (${\mathrm{VO}}_{2}$), carbon dioxide output (${\mathrm{VCO}}_{2}$) and respiration rate (VE) were recorded on a computer system (Vmax 229, Sensor Medics, Anaheim, CA, USA). Aside from resting ${\mathrm{VO}}_{2}$ and peak ${\mathrm{VO}}_{2}$, the gas trades of each patient were also recorded in order to calculate more specific values such as predicted ${\mathrm{VO}}_{2}$ at peak exercise (predicted peak ${\mathrm{VO}}_{2}$), VE/${\mathrm{VCO}}_{2}$ slope, ${\mathrm{VO}}_{2}$ at the anaerobic threshold (AT), peak work rate (WR peak) and work rate at anaerobic threshold (WR at AT). Anaerobic threshold (AT) was determined using the V slope technique during cardiopulmonary exercise testing [8] while the result was graphically confirmed by plotting respiratory equivalent for oxygen (VE/${\mathrm{VO}}_{2}$) and carbon dioxide (VE/${\mathrm{VCO}}_{2}$) simultaneously against time. A 12-lead ECG system was monitoring the heart rate and rhythm of each patient, a pulse oximeter demonstrated saturation and blood pressure was measured every 2 minutes. Termination criteria of CPET included abnormalities in the electrocardiography (ECG), dyspnea, and leg fatigue.

### 2.4 Strength Training Assessment

Strength training assessment was performed by the one-repetition maximum (1RM) test at the beginning and the end of the CR program in all participants. The 1RM test measured each patient’s ability to lift the maximum weight in one repetition. Additionally, muscular endurance test, defined as the maximum number of repetitions at 65% of the weight achieved in the 1RM test, was also recorded. There was always a familiarization day before the test day of the 1RM. The 1RM test was performed the same day with the muscular endurance test. Patients had enough time between the tests in order to recover.

### 2.5 Exercise Training Protocols

Using stratified randomization, patients were randomly allocated in 2 different exercise training groups; either the HIIT or the high-intensity interval training (HIIT) group combined with muscle strength training (COM). The appropriate intensity in watts (W) of each patient’s workout session was structured according to the outcomes of their preliminary CPET. Patients were randomized for age (cut-off point: 50 years) and peak ${\mathrm{VO}}_{2}$ of the initial CPET (cut-off point: 16 mL/kg/min). Then, subjects were identified and assigned into blocks, and simple randomization was performed within each block to assign subjects to one of the groups. Randomization was performed by an investigator not involved in the exercise sessions assessment, and checked twice by 2 independent investigators.

The total duration of each session, as well as the aerobic exercise program, was similar in both groups. Specifically, HIIT was a modified Wisløff’s protocol [38]. All patients performed a 7-minute warm-up on a stationary bike (Ironman M3 Upright Cycle, Keys Fitness Products, LP. Garland, Texas 75041, USA) at 45% of their peak ${\mathrm{VO}}_{2}$, followed by a 3-minute active recovery at 50%. Consecutively, the aerobic session consisted of 4 four-minute sets at 80% of the peak ${\mathrm{VO}}_{2}$, followed by 4 three-minute sets at 50%. There was a gradual increased intensity in the 4 four-minute sets at 80% of the peak ${\mathrm{VO}}_{2}$ by by 5% between the 10th and 18th session, by further 10% between the 19th and 27th session, and by further 10% between the 28th and 36th session. Moreover, intensity was also increased in resistance training by 5% between the 7th and 18th session, by 5% more between the 19th and the 30th session, and by further 5% in the last 6 sessions. The HIIT program lasted 31 minutes in total in both groups.

The main difference between HIIT and combined with strength training (COM) groups was that, at the end of the aerobic exercise, patients of the HIIT group performed balance and coordination exercises including narrow corridor walking, backward narrow corridor walking and side walking in both sides, while patients of the COM group performed resistance training according to the 1RM test including strength exercises for the quadriceps (knee extension), leg curl (knee flexion) and the chest muscles (shoulders flexion & chest press). Strength exercises were initially prescribed to 60% of the 1RM test and then gradually increased in number of repetitions and weight (2–3 sets of 10–12 repetitions between 60%–75% of the 1RM) with 1-minute rest period between sets.

Blood pressure, oxygen saturation and heart rate were measured throughout the session and dyspnea or fatigue was assessed by the Borg scale.

### 2.6 Quality of Life Assessment—Minnesota Living with Heart Failure Questionnaire (MLWHFQ)

Quality of life was evaluated using the validated Greek version of the Minnesota Living with Heart Failure Questionnaire (MLWHFQ) [48], a disease-specific questionnaire originally developed by Rector *et al*. [49] for systematic and comprehensive assessment of patients’ perception of the effects of HF and its management on their daily life. This tool for measuring the functional status of patients with HF includes 21 items consisting of 8 items on physical aspects, 6 on emotional aspects, and 7 other items adding up to the total score that cover socioeconomic and other issues related to HF. The response score ranges from 0–105 points, with higher scores indicating higher severity and, therefore, lower QoL due to HF symptoms.

MLWHF questionnaire was given to each patient prior the start and at the end of the CR program. Patients were given enough time, space and privacy to complete the questionnaire.

### 2.7 Outcome Measures

Primary outcomes of our study were (i) aerobic exercise capacity, including peak ${\mathrm{VO}}_{2}$, assessed by cardiopulmonary exercise testing, (ii) strength exercise capacity of the quadriceps and the chest muscles assessed by 1-repetition maximum test, and (iii) quality of life assessed by the MLWHF questionnaire before and after the completion of the rehabilitation program. Secondary outcomes of the study were (i) muscular endurance of the quadriceps and the chest muscles assessed by the number of repetitions, and (ii) EF.

### 2.8 Statistical Analyses

A power analysis was performed before the initiation of the study, based on previous studies with similar methodology that investigated the improvement in aerobic exercise capacity, through peak ${\mathrm{VO}}_{2}$ and WR at AT, after an exercise training program in patients with CHF. It was estimated that 50 patients, including a percentage of 20% as dropouts, were required in order to observe statistically significant differences after the rehabilitation program with a power level at 0.8 and a level of statistical significance at 0.05.

Normality of distribution was checked with the Shapiro-Wilk test. Variables are expressed as mean ± standard deviation (SD) or median (25th–75th percentiles). Paired two sample student *t*-test analyzed differences of parameters with normal distribution, while Wilcoxon signed-rank test analyzed differences for nonparametric data within total sample and within each exercise group. Unadjusted differences between exercise groups were assessed with factorial analysis of variance (ANOVA) 2 × 2 (time × group). All tests were two tailed and statistical analyses were performed with IBM SPSS 25 (IBM Corp., Chicago, IL, USA) statistics package.

## 3. Results

Demographics and CPET indexes of total sample and within each exercise training group are demonstrated in Table 1 HIIT and COM groups had similar baseline values before the CR program. Patients of both exercise training groups usually reached 13–15 in Borg scale during maximal exercise. HIIT was mostly at 13–14 while COM at 14–15. However, there were no significant differences between groups.

**Table 1.** **Demographic characteristics of all patients with CHF enrolled in the cardiac rehabilitation program and of each exercise training group**.

Demographic characteristics*		All patients	HIIT group	COM group
Number of patients (N)		44	19	25
Gender (Males/Females*)*		35/9	16/3	19/6
Age ${\mathrm{(years)}}^{a}$		56 ± 10	54 ± 11	57 ± 9
Height ${\mathrm{(cm)}}^{a}$		175.2 ± 9.7	176.1 ± 8.4	174.4 ± 10.9
Weight ${\mathrm{(kg)}}^{a}$		89.1 ± 23.4	92.2 ± 24.0	86.3 ± 23.1
BMI (kg/$m^{2}$${)}^{a}$		28.7 ± 5.2	29.4 ± 6.0	28.1 ± 4.7
NYHA stage (class II/III)		34/10	14/5	20/5
EF before rehabilitation ${\mathrm{(\%)}}^{b}$		30 (28–40)	35 (30–42.5)	30 (25–35)
Type of CHF				
	Dilated cardiomyopathy [n (%)]	12 (27.3%)	4 (21%)	8 (32%)
	Ischemic [n (%)]	24 (54.5%)	11 (58%)	13 (52%)
	Other (valvopathy, etc.) [n (%)]	8 (18.2%)	4 (21%)	4 (16%)
Medication				
	Diuretics [n (%)]	29 (66%)	12 (63%)	17 (68%)
	ACE inhibitors [n (%)]	22 (50%)	10 (53%)	12 (48%)
	ARBs [n (%)]	5 (11%)	4 (21%)	1 (4%)
	b Blockers [n (%)]	43 (98%)	19 (100%)	24 (96%)
	Aldosterone Antagonists [n (%)]	32 (73%)	14 (74%)	18 (72%)
	Ca blockers [n (%)]	1 (2%)	0 (0%)	1 (4%)
	Vasodilators [n (%)]	2 (5%)	1 (5%)	1 (4%)
	Digoxin [n (%)]	3 (7%)	0 (0%)	3 (12%)
	Amiodarone [n (%)]	6 (14%)	3 (16%)	3 (12%)
Cardiopulmonary exercise testing and muscular indexes before rehabilitation*				
	Rest ${\mathrm{VO}}_{2}$ ${\mathrm{(mL/kg/min)}}^{a}$	4.7 ± 0.9	4.7 ± 0.8	4.7 ± 1.1
	Peak ${\mathrm{VO}}_{2}$ ${\mathrm{(mL/kg/min)}}^{a}$	18.4 ± 4.4	18.4 ± 5.0	18.5 ± 3.9
	Peak predicted ${\mathrm{VO}}_{2}$ ${\mathrm{(\%)}}^{a}$	64 ± 15	62.2 ± 19.6	65.4 ± 11.5
	${\mathrm{VE/VCO}}_{2}$ slope	29 ± 5	28.5 ± 6.8	29.4 ± 3.4
	Peak WR ${\mathrm{(watts)}}^{a}$	101 ± 39	103.6 ± 40	99± 38.2
	Relative peak WR ${\mathrm{(watts/kg of body weight)}}^{a}$	1.14 ± 0.36	1.15 ± 0.43	1.14 ± 0.31
	WR at AT ${\mathrm{(watts)}}^{a}$	42.9 ± 20.2	46.1 ± 19.1	40.5 ± 21.1
	Relative WR at AT ${\mathrm{(watts/kg of body weight)}}^{a}$	0.49 ± 0.21	0.52 ± 0.22	0.47 ± 0.21
	1RM quadriceps ${\mathrm{(kg)}}^{b}$	42 (30–53)	47 (28–52)	40 (31–54)
	Relative 1RM quadriceps ${\mathrm{(kg/kg of body weight)}}^{b}$	0.49 (0.37–0.57)	0.51 (0.35–0.59)	0.47 (0.41–0.55)
	1RM chest muscles ${\mathrm{(kg)}}^{b}$	50 (35–70)	60 (45–75)	45 (28–68)
	Relative 1RM chest muscles ${\mathrm{(kg/kg of body weight)}}^{b}$	0.60 (0.42–0.79)	0.75 (0.48–0.82)	0.56 (0.40–0.70)
	Muscular endurance quadriceps ${\mathrm{(repetitions)}}^{b}$	10 (8–12)	10 (7–13)	10 (8–11)
	Muscular endurance chest muscles ${\mathrm{(repetitions)}}^{b}$	12 (10–16)	12 (10–16)	11 (10–16)

BMI, body mass index; HIIT group, high-intensity interval exercise group; COM group, HIIT combined with muscle strength exercise group; NYHA, New York Heart Association; CHF, chronic heart failure; ACE, angiotensin-converting-enzyme; ARB, angiotensin II receptor blockers; ${\mathrm{VO}}_{2}$, oxygen uptake; ${\mathrm{VCO}}_{2}$, carbon dioxide output; WR, work rate; RM, repetition maximum; ${\mathrm{VE/VCO}}_{2}$ slope, the slope of the ventilatory equivalent for carbon dioxide; Peak WR, peak work rate; WR at AT, the workload at the anaerobic threshold; 1RM, one-repetition maximum; CPET, cardiopulmonary exercise testing; EF, ejection fraction; SD, standard deviation. ^a^Values are expressed as mean ± SD; ^b^Values are expressed as median (25th–75th percentiles); *There was no statistically significant difference between the two exercise training groups for demographic characteristics, medication, and baseline CPET and muscular test indices (*p *> 0.05).

As far as the total number of CHF patients is concerned, the cardiac rehabilitation program had beneficial effects on their functional capacity, quality of life and ejection fraction. Most specifically, most CPET indexes including peak ${\mathrm{VO}}_{2}$, predicted peak ${\mathrm{VO}}_{2}$, ${\mathrm{VE/VCO}}_{2}$ slope, ${\mathrm{VO}}_{2}$ at AT, peak WR, peak VE, WR at AT and peak $P_{\text{ET}}$${\mathrm{CO}}_{2}$ improved after the CR program (**Supplementary Table 1**). Quality of life of CHF patients improved, as physical, emotional and total score of the MLHFQ decreased after rehabilitation (**Supplementary Table 1**). Finally, indexes of muscle function such as the 1RM test and muscular endurance were increased after rehabilitation (**Supplementary Table 1**).

The beneficial effect of the CR program was also shown within each exercise training group. Patients in HIIT group improved rest ${\mathrm{VO}}_{2}$, peak ${\mathrm{VO}}_{2}$, predicted peak ${\mathrm{VO}}_{2}$, peak WR, ${\mathrm{HRR}}_{1}$, peak VE, WR at AT and peak $P_{\text{ET}}$${\mathrm{CO}}_{2}$ from CPET indexes, physical and total score of MLHFQ, 1RM test and muscular endurance and their EF (Table 2). Similarly, patients in COM group improved predicted peak ${\mathrm{VO}}_{2}$, AT, peak WR, peak VE and peak WR at AT from CPET indexes, physical and total score of MLHFQ, 1RM and muscular endurance, as well as their EF (Table 2). However, improvement in most variables was similar between HIIT and COM group, indicating the benefits of the exercise training program regardless of their protocol (Table 2). Nevertheless, there were differences in some variables. Specifically, the improvement in the 1RM test, number of repetitions of chest muscles (muscular endurance) and peak work rate at AT was higher in COM group in comparison with HIIT (Table 2).

**Table 2.** **Changes in various variables observed within and between the two exercise training groups after the cardiac rehabilitation program (after CR) compared with the baseline values (before CR)**.

Variables of the CR program		HIIT group (19 patients)			COM group (25 patients)			*p* value between groups
		Before CR	After CR	*p* value*	Before CR	After CR	*p* value*	
Minnesota living with heart failure quality of life questionnaire								
	Physical score (units)	11 (5–25)	9 (2–16)	0.020	10 (5–21)	5 (2–9)	0.001	0.962
	Emotional score (units)	7 (3–14)	4 (1–9)	0.131	5 (2–9)	3 (1–8)	0.188	0.557
	Total score (units)	33 (16–49)	21 (7–31)	0.017	23 (13–46)	12 (7–24)	0.004	0.704
1 Repetition maximum ${\mathrm{test}}^{a}$								
	Quadriceps (kg)	47 (28–52)	57 (47–65)	<0.001	40 (31–54)	52 (34–63)	<0.001	0.785
	Relative 1RM quadriceps (kg/kg of body weight)	0.51 (0.35–0.59)	0.60 (0.48–0.72)	<0.001	0.47 (0.41–0.55)	0.55 (0.49–0.71)	<0.001	0.767
	Chest muscles (kg)	60 (45–75)	65 (50–85)	0.006	45 (28–68)	55 (41–88)	<0.001	0.039
	Relative 1RM chest muscles (kg/kg of body weight)	0.75 (0.48–0.82)	0.79 (0.56–0.91)	0.007	0.56 (0.40–0.70)	0.71 (0.57–0.88)	<0.001	0.021
Muscular ${\mathrm{endurance}}^{a}$								
	Quadriceps (repetitions)	10 (7–13)	15 (10–20)	<0.001	10 (8–11)	13 (12–15)	<0.001	0.294
	Chest muscles (repetitions)	12 (10–16)	17 (12–20)	<0.001	11 (10–16)	19 (15–26)	<0.001	0.002
Cardiopulmonary exercise testing indexes								
	Rest ${\mathrm{VO}}_{2}$ (mL/kg/min)	4.7 ± 0.8	4.1 ± 1.0	0.025	4.7 ± 1.1	4.5 ± 1.2	0.518	0.312
	Peak ${\mathrm{VO}}_{2}$ (mL/kg/min)	18.4 ± 5.0	21.5 ± 7.3	0.011	18.5 ± 3.9	20.1 ± 4.3	0.071	0.290
	Predicted peak ${\mathrm{VO}}_{2}$ (%)	62.2 ± 19.6	73.6 ± 28.0	0.006	65.4 ± 11.5	72.2 ± 15.8	0.028	0.322
	${\mathrm{VE/VCO}}_{2}$ slope	28.5 ± 6.8	26.4 ± 5.5	0.101	29.4 ± 3.4	28.6 ± 5.2	0.260	0.380
	AT (mL/kg/min)	12.1 ± 2.6	12.9 ± 2.3	0.093	11.7 ± 2.7	13.7 ± 3.3	0.003	0.101
	Peak WR (watts)	103.6 ± 40.0	122.2 ± 43.5	<0.001	99.0 ± 38.2	119.1 ± 46.1	<0.001	0.811
	Relative peak WR (watts/kg of body weight)	1.15 ± 0.43	1.35 ± 0.47	<0.001	1.14 ± 0.31	1.36 ± 0.34	<0.001	0.704
	RQ/RER	1.3 ± 0.4	1.2 ± 0.2	0.344	1.2 ± 0.2	1.1 ± 0.2	0.383	0.462
	Peak VE (L/min)	46.3 ± 14.6	62.9 ± 18.4	<0.001	53.1 ± 23.0	70.5 ± 25.2	<0.001	0.861
	WR at AT (watts)	46.1 ± 19.1	65.8 ± 23.6	<0.001	40.5 ± 21.1	69.8 ± 26.3	<0.001	0.023
	Relative WR at AT (watts/kg of body weight)	0.52 ± 0.22	0.74 ± 0.27	<0.001	0.47 ± 0.21	0.80 ± 0.21	<0.001	0.002
	Peak $P_{\text{ET}}$${\mathrm{CO}}_{2}$ (mmHg)	39.8 ± 8.4	33.7 ± 6.2	0.007	34.8 ± 7.6	33.5 ± 4.2	0.400	0.062
Ultrasound indexes								
	Ejection fraction (%)	35 (30–42)	35 (30–45)	0.004	30 (25–35)	35 (30–40)	0.001	0.587

AT, anaerobic threshold; HIIT group, high-intensity interval exercise group; COM group, HIIT combined with muscle strength exercise group; CR, cardiac rehabilitation; Rest ${\mathrm{VO}}_{2}$, oxygen uptake at rest; Peak ${\mathrm{VO}}_{2}$, peak oxygen uptake; Predicted peak ${\mathrm{VO}}_{2}$, predicted peak oxygen uptake; ${\mathrm{VE/VCO}}_{2}$ slope, the slope of the ventilatory equivalent for carbon dioxide; AT, anaerobic threshold; Peak WR, peak work rate; RQ, respiratory quotient; RER, respiratory exchange ratio; Peak VE, peak minute ventilation; WR at AT, the workload at the anaerobic threshold; $P_{\text{ET}}$${\mathrm{CO}}_{2}$, end-tidal partial pressure of ${\mathrm{CO}}_{2}$; EF, ejection fraction; SD, standard deviation. ^a^Values are presented as median (25th–75th percentiles); ^b^Values are presented as mean ± SD; *p-value for differences in variables within each exercise training group.

## 4. Discussion

It has been previously shown that aerobic exercise training, and spesifically HIIT, has a positive impact on functional capacity, muscular endurance, and QoL in CHF patients. This study demonstrated that the addition of muscle strength training to a HIIT protocol (COM) results in improved workload at the anaerobic threshold and better performance in the 1RM and muscular endurance test of the chest muscles in CHF patients. Overall, it appears that COM has some advantages regarding aerobic capacity and muscle strength improvement when compared to HIIT protocol. However, the comparison between HIIT and COM regarding other indices of CPET and quality of life, revealed similar improvements in both groups, suggesting that the addition of strength training to HIIT may not have a cumulative beneficial effect.

During the last decades, many studies have evaluated the effect of HIIT on functional capacity, microcirculation, vascular endothelial and skeletal muscle function, and QoL in CHF patients, demonstrating beneficial effects on most of these parameters [19, 20, 21, 22, 23, 24, 25, 26, 27, 28, 29, 30, 31, 32, 33, 34, 35, 36, 37, 38, 39, 40, 41, 42]. Moreover, the superior effects of HIIT protocols in CHF patients undergoing rehabilitation compared to continuous or moderate aerobic training have been also shown [28, 32, 33, 34, 35, 36]. However, data regarding the effects of combined exercise training protocols such as HIIT with strength training is limited in literature. There are only a few studies that compared HIIT versus HIIT and muscle strength training in order to evaluate the impact of the addition of resistant training (RT) on functional capacity, physical performance and QoL [24, 28, 39, 40, 41, 42].

Specifically, Bouchla *et al*. [28] investigated the additional effects of strength training on muscle strength, functional capacity and body composition in CHF patients participating in an interval aerobic training program. They showed a greater improvement in the combined training than in the aerobic training group regarding the 2RM test. However, there was no difference in total lean mass, total fat mass, leg lean mass, leg fat mass, and CPET indices such as peak ${\mathrm{VO}}_{2}$, ${\mathrm{VO}}_{2}$ at AT, peak work rate and work rate at AT between the two groups. Similarly, Anagnostakou *et al*. [40] compared the effects of interval cycle training combined with strength training versus interval training alone on vascular reactivity in CHF patients. Authors observed a significant improvement in flow-mediated vasodilation (FMD) and the 2RM test in the combined group compared to the interval training group, without revealing any other significant changes in CPET parameters, including peak ${\mathrm{VO}}_{2}$, peak work rate and ${\mathrm{VO}}_{2}$ at AT. In addition, Georgantas *et al*. [39] investigated the effects of HIIT compared with combined exercise training on early ventilatory and metabolic recovery pattern after a symptom-limited CPET, following a 3-month CR program in CHF patients. The combined training group showed a greater improvement in VE and respiratory rate during the first minute of recovery after exercise compared with the HIIT group. However, other indices of functional capacity such as peak ${\mathrm{VO}}_{2}$, ${\mathrm{VO}}_{2}$ at AT, peak work rate, and ${\mathrm{VE/VCO}}_{2}$ slope did not show differences between HIIT and combined groups. Finally, Agapitou *et al*. [24] investigated the effects of incorporating resistance training in aerobic interval training on exercise capacity and circulating levels of anabolic factors in CHF patients. Authors showed that the combination of aerobic and resistance training was superior to aerobic training alone regarding CPET indices including peak ${\mathrm{VO}}_{2}$, peak work rate, and 2RM test for the quadriceps, presenting also a trend for improvement of anabolic steroid concentration in these patients.

Some of our findings may differ compared to the results of previous studies, as we observed significant improvements in the work rate at AT, the 1RM test and muscular endurance of the chest muscles and we did not manage to demonstrate differences in other CPET or QoL indices. A possible explanation of these differences is that, in the present study, we used exercise protocols characterized by different intensity, workload, active recovery intervals and types of RT compared with the previous studies. Moreover, our patients had already good functional capacity at baseline, being of low or intermediate HF severity. It is worth mentioning that our study evaluated another significant parameter, EF. We found that EF improved within each exercise training group after the CR program.

In this study, we examined all aspects of the MLWHF questionnaire (physical, emotional, and total scores) revealing that patients improved in physical and total score in both groups. However, the emotional aspect didn’t improve in either group despite the beneficial effects of exercise training on the patients’ cardiovascular system and functional capacity. This may suggest that further psychological evaluation is needed at baseline and during the exercise training program, as psychopathological symptoms, such as anxiety or depression, are common in CHF patients [50, 51].

Aerobic and resistance exercise training result in beneficial effects on skeletal muscles, inducing skeletal muscle hypertrophy, reversal of the altered muscle fiber composition and increase in mitochondrial and capillary density in CHF patients [19, 40, 52]. Indeed, in the present study, patients in both HIIT and COM exercise training groups improved their strength indexes, including the 1RM test and muscular endurance. In addition, the comparison between HIIT and COM revealed improvements in the upper extremities muscle function indexes (1RM and muscular endurance) in favor of the COM group. However, we did not observe differences in the lower extremities muscle function. This finding could be explained by the fact that patients of both group performed cycling exercise training, which might have improved skeletal myopathy in a similar way in both HIIT and COM groups. The improvement in muscular strength of the lower limbs and exercise tolerance in patients of both groups might be associated with the exercise-induced increase in oxygen extraction and the improvement of peak ${\mathrm{VO}}_{2}$ and EF that occurred in both groups. Thus, the addition of RT to a cycling-based HIIT protocol in CHF patients does not appear to have cumulative benefits in the lower extremities muscle function of those patients.

Finally, as far as safety of a cardiopulmonary rehabilitation program is concerned, there is a previous study by Ellingsen Ø *et al*. [53] where authors reported 9 severe cardiovascular and 6 non-cardiovascular events during the exercise training program, as well as 19 severe cardiovascular and 3 non-cardiovascular events at follow-up within the first year. Among cardiovascular events, 2 were fatal at follow-up while none of them was fatal during the program. Among non-cardiovascular events, there was 1 fatal event at follow-up and no fatal events during exercise. It is also noteworthy that the majority of adverse events happened in patients who underwent HIIT (39%) compared to moderate continuous training (34%) and recommended regular exercise (25%). In our study, no severe cardiovascular or non-cardiovascular adverse events during our rehabilitation program were observed. Rehospitalizations due to non-cardiovascular events occurred in 3 patients out of the 44 of our study at 1-year follow-up. In most studies in literature, HIIT has been shown to be safe and feasible in patients with HF and other populations [54, 55, 56, 57]. Moreover, combined training, and specifically resistance training after HIIT or moderate intensity continuous training, has also been proven to be feasible, even in untrained older adults [58]. In our study, all patients of the COM group successfully completed resistance training after HIIT in each session. Consequently, combined exercise training regimes seem to be feasible and safe in patients with heart failure and reduced or mildly-reduced ejection fraction.

There are some important limitations. The small number of the study participants might have led to underpowered comparisons between groups for some indices that did not allow revealing additional possible benefits of COM in the parameters of interest.

Our study presented the beneficial effects of muscle strength training in combination with HIIT-based aerobic exercise training. Although randomized controlled studies combining RT and HIIT and including larger numbers of CHF patients are required in order to reveal potentially more beneficial effects of exercise training on functional ca-pacity and vascular endothelial function of those patients, the addition of individualized RT to aerobic exercise should be included in cardiac rehabilitation programs of CHF patients. RT has been found to improve skeletal muscle mass and function, and reverse skeletal myopathy [19, 27, 28] which are both characteristics in heart failure, leading in reduced functional capacity and poor QoL. We supposed that the increase in the 1RM test and in muscular endurance, even in the chest muscles only, may contribute to the reverse of skeletal myopathy which, in our opinion, is one of the most important issues in HF. On the other hand, patients performing HIIT alone may improve their balance compared to COM, which may also be important aspect in HF. However, this was not a parameter of interest in our study and, unfortunately, we did not compare it between the 2 groups. Finally, the addition of other exercise interventions to the HIIT and RT exercise protocols, such as breathing exercises and inspiratory muscle training might be also an interesting field for future research focusing on the exercise-induced functional and clinical adaptations in CHF patients.

## 5. Conclusions

The addition of muscle strength training to a HIIT protocol resulted in better workload at the AT, as well as in improvement of the 1RM test and muscular endurance of the chest muscles in CHF patients. Our findings further suggest that implementing resistance training in HIIT protocols within the CR programs may result in greater exercise-induced benefits. Improvements in muscular function parameters such as the 1RM test and muscular endurance, may improve skeletal myopathy of CHF patients leading, thus, in better functional capacity and improved QoL. However, further studies are required to uncover potential mechanisms of exercise-induced beneficial adaptations in patients with heart failure.

## Data Availability

The data that support the findings of this study are available on request from the corresponding author [MA]. The data are not puplically available due to their containing information that could compromise the privacy of research participants.

## Abbreviations

CR, cardiac rehabilitation; HIIT group, high-intensity interval exercise group; COM group, HIIT combined with muscle strength exercise group; NYHA, New York Heart Association; CHF, chronic heart failure; ACE, angiotensin-converting-enzyme; ARB, angiotensin II receptor blockers; ${\mathrm{VCO}}_{2}$, carbon dioxide output; CPET, cardiopulmonary exercise testing; ${\mathrm{VO}}_{2}$, oxygen uptake; ${\mathrm{VE/VCO}}_{2}$ slope, the slope of the ventilatory equivalent for carbon dioxide; AT, anaerobic threshold; WR, work rate; RQ, respiratory quotient; RER, respiratory exchange ratio; VE, minute ventilation; $P_{\text{ET}}$${\mathrm{CO}}_{2}$, end-tidal partial pressure of ${\mathrm{CO}}_{2}$; EF, ejection fraction; SD, standard deviation.

## Supplementary Material

Supplementary material associated with this article can be found, in the online version, at https://doi.org/10.31083/j.rcm2401029.

## References

[1] Ponikowski P, Voors AA, Anker SD, Bueno H, Cleland JG, Coats AJ (2016). 2016 ESC Guidelines for the diagnosis and treatment of acute and chronic heart failure: The Task Force for the diagnosis and treatment of acute and chronic heart failure of the European Society of Cardiology (ESC) Developed with the special contribution of the Heart Failure Association (HFA) of the ESC. *European Heart Journal*.

[2] Mosterd A, Hoes AW (2007). Clinical epidemiology of heart failure. *Heart*.

[3] Virani SS, Alonso A, Aparicio HJ, Benjamin EJ, Bittencourt MS, Callaway CW (2021). Heart disease and stroke statistics-2021 update: a report from the American Heart Association. *Circulation*.

[4] Ponikowski P, Anker SD, AlHabib KF, Cowie MR, Force TL, Hu S (2014). Heart failure: preventing disease and death worldwide. *ESC Heart Failure*.

[5] Manetos C, Dimopoulos S, Tzanis G, Vakrou S, Tasoulis A, Kapelios C (2011). Skeletal muscle microcirculatory abnormalities are associated with exercise intolerance, ventilatory inefficiency, and impaired autonomic control in heart failure. *The Journal of Heart and Lung Transplantation*.

[6] Tikhomirova I, Petrochenko E, Muravyov A, Malysheva Y, Petrochenko A, Yakusevich V (2017). Microcircu-lation and blood rheology abnormalities in chronic heart failure. *Clinical Hemorheology and Microcirculation*.

[7] Sidik N, Morrow A, Berry C (2020). Human Microcirculation in Ischemic Heart Disease. *Arteriosclerosis, Thrombosis, and Vascular Biology*.

[8] Tzanis G, Manetos C, Dimopoulos S, Vasileiadis I, Malliaras K, Kaldara E (2016). Attenuated Microcirculatory Response to Maximal Exercise in Patients with Chronic Heart Failure. *Journal of Cardiopulmonary Rehabilitation and Prevention*.

[9] Del Buono MG, Arena R, Borlaug BA, Carbone S, Canada JM, Kirkman DL (2019). Exercise Intolerance in Patients with Heart Failure: JACC State-of-the-Art Review. *Journal of the American College of Cardiology*.

[10] Poole DC, Richardson RS, Haykowsky MJ, Hirai DM, Musch TI (2018). Exercise limitations in heart failure with reduced and preserved ejection fraction. *Journal of Applied Physiology*.

[11] Tzanis G, Dimopoulos S, Agapitou V, Nanas S (2014). Exercise intolerance in chronic heart failure: the role of cortisol and the catabolic state. *Current Heart Failure Reports*.

[12] Agapitou V, Dimopoulos S, Kapelios C, Karatzanos E, Manetos C, Georgantas A (2013). Hormonal imbalance in relation to exercise intolerance and ventilatory inefficiency in chronic heart failure. *The Journal of Heart and Lung Transplantation*.

[13] Springer J, Springer J, Anker SD (2017). Muscle wasting and sarcopenia in heart failure and beyond: update 2017. *ESC Heart Failure*.

[14] Lavine KJ, Sierra OL (2017). Skeletal muscle inflammation and atrophy in heart failure. *Heart Failure Reviews*.

[15] Yokota T, Kinugawa S, Hirabayashi K, Yamato M, Takada S, Suga T (2021). Systemic oxidative stress is associated with lower aerobic capacity and impaired skeletal muscle energy metabolism in heart failure patients. *Scientific Reports*.

[16] Philippou A, Xanthis D, Chryssanthopοulos C, Maridaki M, Koutsilieris M (2020). Heart Failure-Induced Skeletal Muscle Wasting. *Current Heart Failure Reports*.

[17] Johansson I, Joseph P, Balasubramanian K, McMurray JJV, Lund LH, Ezekowitz JA (2021). Health-Related Quality of Life and Mortality in Heart Failure: the Global Congestive Heart Failure Study of 23 000 Patients from 40 Countries. *Circulation*.

[18] Heo S, Lennie TA, Okoli C, Moser DK (2009). Quality of life in patients with heart failure: ask the patients. *Heart Lung*.

[19] Tzanis G, Philippou A, Karatzanos E, Dimopoulos S, Kaldara E, Nana E (2017). Effects of High-Intensity Interval Exercise Training on Skeletal Myopathy of Chronic Heart Failure. *Journal of Cardiac Failure*.

[20] Tryfonos A, Tzanis G, Pitsolis T, Karatzanos E, Koutsilieris M, Nanas S (2021). Exercise Training Enhances Angio-genesis-Related Gene Responses in Skeletal Muscle of Patients with Chronic Heart Failure. *Cells*.

[21] Kourek C, Karatzanos E, Psarra K, Ntalianis A, Mitsiou G, Delis D (2021). Endothelial progenitor cells mobilization after maximal exercise in patients with chronic heart failure. *Hellenic Journal of Cardiology*.

[22] Kourek C, Alshamari M, Mitsiou G, Psarra K, Delis D, Linardatou V (2020). The acute and long-term effects of a cardiac rehabilitation program on endothelial progenitor cells in chronic heart failure patients: Comparing two different exercise training protocols. *International Journal of Cardiology Heart & Vasculature*.

[23] Kourek C, Karatzanos E, Psarra K, Georgiopoulos G, Delis D, Linardatou V (2020). Endothelial progenitor cells mobilization after maximal exercise according to heart failure severity. *World Journal of Cardiology*.

[24] Agapitou V, Tzanis G, Dimopoulos S, Karatzanos E, Karga H, Nanas S (2018). Effect of combined endurance and resistance training on exercise capacity and serum anabolic steroid concentration in patients with chronic heart failure. *Hellenic Journal of Cardiology*.

[25] Kitzman DW, Brubaker P, Morgan T, Haykowsky M, Hundley G, Kraus WE (2016). Effect of Caloric Restriction or Aerobic Exercise Training on Peak Oxygen Consumption and Quality of Life in Obese Older Patients with Heart Failure with Preserved Ejection Fraction: A Randomized Clinical Trial. *The Journal of the American Medical Association*.

[26] Kitzman DW, Whellan DJ, Duncan P, Pastva AM, Mentz RJ, Reeves GR (2021). Physical Rehabilitation for Older Patients Hospitalized for Heart Failure. *The New England Journal of Medicine*.

[27] Hirai DM, Musch TI, Poole DC (2015). Exercise training in chronic heart failure: improving skeletal muscle O2 transport and utilization. *American Journal of Physiology-Heart and Circulatory Physiology*.

[28] Bouchla A, Karatzanos E, Dimopoulos S, Tasoulis A, Agapitou V, Diakos N (2011). The Addition of Strength Training to Aerobic Interval Training. *Journal of Cardiopulmonary Rehabilitation and Prevention*.

[29] Taylor RS, Walker S, Smart NA, Piepoli MF, Warren FC, Ciani O (2019). Impact of Exercise Rehabilitation on Exercise Capacity and Quality-of-Life in Heart Failure: Individual Participant Meta-Analysis. *Journal of the American College of Cardiology*.

[30] Dallas K, Dinas PC, Chryssanthopoulos C, Dallas G, Maridaki M, Koutsilieris M (2021). The effects of exercise on VO2peak, quality of life and hospitalization in heart failure patients: A systematic review with meta-analyses. *European Journal of Sport Science*.

[31] McDonagh TA, Metra M, Adamo M, Gardner RS, Baumbach A, Böhm M (2021). 2021 ESC Guidelines for the diagnosis and treatment of acute and chronic heart failure. *European Heart Journal*.

[32] Gomes Neto M, Durães AR, Conceição LSR, Saquetto MB, Ellingsen Ø, Carvalho VO (2018). High intensity interval training versus moderate intensity continuous training on exercise capacity and quality of life in patients with heart failure with reduced ejection fraction: A systematic review and meta-analysis. *International Journal of Cardiology*.

[33] Benda NM, Seeger JP, Stevens GG, Hijmans-Kersten BT, van Dijk AP, Bellersen L (2015). Effects of High-Intensity Interval Training versus Continuous Training on Physical Fitness, Cardiovascular Function and Quality of Life in Heart Failure Patients. *PLoS ONE*.

[34] Angadi SS, Mookadam F, Lee CD, Tucker WJ, Haykowsky MJ, Gaesser GA (2015). High-intensity interval training vs. moderate-intensity continuous exercise training in heart failure with preserved ejection fraction: a pilot study. *Journal of Applied Physiology*.

[35] Freyssin C, Verkindt C, Prieur F, Benaich P, Maunier S, Blanc P (2012). Cardiac Rehabilitation in Chronic Heart Failure: Effect of an 8-Week, High-Intensity Interval Training Versus Continuous Training. *Archives of Physical Medicine and Rehabilitation*.

[36] Nechwatal RM, Duck C, Gruber G (2002). Physical training as interval or continuous training in chronic heart failure for improving functional capacity, hemodynamics and quality of life–a controlled study. *Z Kardiol*.

[37] Ellingsen Ø, Halle M, Conraads V, Støylen A, Dalen H, Delagardelle C (2017). SMARTEX Heart Failure Study (Study of Myocardial Recovery After Exercise Training in Heart Failure) Group. High-Intensity Interval Training in Patients with Heart Failure with Reduced Ejection Fraction. *Circulation*.

[38] Wisløff U, Støylen A, Loennechen JP, Bruvold M, Rognmo Ø, Haram PM (2007). Superior Cardiovascular Effect of Aerobic Interval Training Versus Moderate Continuous Training in Heart Failure Patients. *Circulation*.

[39] Georgantas A, Dimopoulos S, Tasoulis A, Karatzanos E, Pantsios C, Agapitou V (2014). Beneficial effects of combined exercise training on early recovery cardiopulmonary exercise testing indices in patients with chronic heart failure. *Journal of Cardiopulmonary Rehabilitation and Prevention*.

[40] Anagnostakou V, Chatzimichail K, Dimopoulos S, Karatzanos E, Papazachou O, Tasoulis A (2011). Effects of interval cycle training with or without strength training on vascular reactivity in heart failure patients. *Journal of Cardiac Failure*.

[41] Adamopoulos S, Schmid J, Dendale P, Poerschke D, Hansen D, Dritsas A (2014). Combined aerobic/inspiratory muscle training vs. aerobic training in patients with chronic heart failure. *European Journal of Heart Failure*.

[42] Beckers PJ, Denollet J, Possemiers NM, Wuyts FL, Vrints CJ, Conraads VM (2008). Combined endurance-resistance training vs. endurance training in patients with chronic heart failure: a prospective randomized study. *European Heart Journal*.

[43] Jewiss D, Ostman C, Smart NA (2016). The effect of resistance training on clinical outcomes in heart failure: a systematic review and meta-analysis. *International Journal of Cardiology*.

[44] Gomes-Neto M, Durães AR, Conceição LSR, Roever L, Silva CM, Alves IGN (2019). Effect of combined aerobic and resistance training on peak oxygen consumption, muscle strength and health-related quality of life in patients with heart failure with reduced left ventricular ejection fraction: A systematic review and meta-analysis. *International Journal of Cardiology*.

[45] Jesus ICD, Menezes Junior FJD, Bento PCB, Wiens A, Mota J, Leite N (2020). Effect of combined interval training on the cardiorespiratory fitness in heart failure patients: a systematic review and meta-analysis. *Brazilian Journal of Physical Therapy*.

[46] Ambrosetti M, Abreu A, Corrà U, Davos CH, Hansen D, Frederix I (2021). Secondary prevention through comprehensive cardiovascular rehabilitation: From knowledge to implementation. 2020 update. A position paper from the Secondary Prevention and Rehabilitation Section of the European Association of Preventive Cardiology. *European Journal of Preventive Cardiology*.

[47] Hansen JE, Sue DY, Wasserman K (1984). Predicted values for clinical exercise testing. *The American Review of Respiratory Disease*.

[48] Lambrinou E, Kalogirou F, Lamnisos D, Papathanassoglou E, Protopapas A, Sourtzi P (2014). The Greek version of the 9-item European heart failure self-care behaviour scale: a multidimensional or a uni-dimensional scale. *Heart & Lung*.

[49] Rector TS, Kubo SH, Cohn JN (1987). PatientL’s self-assessment of their congestive heart failure: Content, reliability, and validity of a new measure: The Minnesota living with heart failure questionnaire. *Heart Failure*.

[50] Aggelopoulou Z, Fotos NV, Chatziefstratiou AA, Giakoumidakis K, Elefsiniotis I, Brokalaki H (2017). The level of anxiety, depression and quality of life among patients with heart failure in Greece. *Applied Nursing Research*.

[51] Chen X, Xin Y, Hu W, Zhao Y, Zhang Z, Zhou Y (2019). Quality of life and outcomes in heart failure patients with ejection fractions in different ranges. *PLoS ONE*.

[52] Taya M, Amiya E, Hatano M, Maki H, Nitta D, Saito A (2018). Correction to: High-intensity aerobic interval training can lead to improvement in skeletal muscle power among in-hospital patients with advanced heart failure. *Heart Vessels*.

[53] Ellingsen Ø, Halle M, Conraads V, Støylen A, Dalen H, Delagardelle C (2017). High-Intensity Interval Training in Patients With Heart Failure With Reduced Ejection Fraction. *Circulation*.

[54] Pires Peixoto R, Trombert V, Poncet A, Kizlik J, Gold G, Ehret G (2020). Feasibility and safety of high-intensity interval training for the rehabilitation of geriatric inpatients (HIITERGY) a pilot randomized study. *BMC Geriatrics*.

[55] Taylor JL, Holland DJ, Keating SE, Leveritt MD, Gomersall SR, Rowlands AV (2020). Short-term and Long-term Feasibility, Safety, and Efficacy of High-Intensity Interval Training in Cardiac Rehabilitation. *JAMA Cardiology*.

[56] Alvarez Villela M, Chinnadurai T, Salkey K, Furlani A, Yanamandala M, Vukelic S (2021). Feasibility of high-intensity interval training in patients with left ventricular assist devices: a pilot study. *ESC Heart Failure*.

[57] Wang C, Xing J, Zhao B, Wang Y, Zhang L, Wang Y (2022). The Effects of High-Intensity Interval Training on Exercise Capacity and Prognosis in Heart Failure and Coronary Artery Disease: A Systematic Review and Meta-Analysis. *Cardiovascular Therapeutics*.

[58] Falck RS, Davis JC, Milosevic E, Liu-Ambrose T (2017). How much will older adults exercise? A feasibility study of aerobic training combined with resistance training. *Pilot and Feasibility Studies*.

