# Supplementary material for: Does the Addition of Strength Training to a High-Intensity Interval Training Program Benefit More the Patients with Chronic Heart Failure
Source: Rev Cardiovasc Med. 2023 Jan 16;24(1):29. doi: 10.31083/j.rcm2401029 (PMC11270399; doi:10.31083/j.rcm2401029)
Supplement: Supplementary file 1 [file 2153-8174-24-1-029-s1.zip › 2153-8174-24-1-029-s1.docx]

| **Supplementary Table 1. Changes in various variables observed after the cardiac rehabilitation (after CR) program compared with the baseline values (before CR) in all patients (n=44) with chronic heart failure.** | | | |
| --- | --- | --- | --- |
| **Variables of the CR program** | **Before CR** | **After CR** | **P-value** |
| **Minnesota Living with Heart Failure Questionnaire^a^** | | | |
| Physical score (units) | 11 (5 - 22) | 6 (2 - 12) | **< 0.001** |
| Emotional score (units) | 6 (2 - 10) | 4 (1 - 8) | **0.035** |
| Total score (units) | 28 (13 - 48) | 14 (7 - 29) | **< 0.001** |
| **1 Repetition Maximum test^a^** | | | |
| Quadriceps (kg) | 42 (30 - 53) | 55 (40 - 65) | **< 0.001** |
| Relative 1RM quadriceps (kg/kg of body weight) | 0.49 (0.37 - 0.57) | 0.59 (0.49 - 0.71) | **< 0.001** |
| Chest muscles (kg) | 50 (35 - 70) | 64 (43 - 85) | **< 0.001** |
| Relative 1RM chest muscles (kg/kg of body weight) | 0.60 (0.42 -0.79) | 0.74 (0.56 -0.90) | **< 0.001** |
| **Muscular Endurance^a^** | | | |
| Quadriceps (repetitions) | 10 (8 - 12) | 13 (11 - 16) | **< 0.001** |
| Chest muscles (repetitions) | 12 (10 - 16) | 17 (14 - 21) | **< 0.001** |
| **Cardiopulmonary exercise testing indexes^b^** | | | |
| Rest VO_2_ (ml/kg/min) | 4.7 ± 1.0 | 4.3 ± 1.1 | **0.021** |
| Peak VO_2_ (ml/kg/min) | 18.4 ± 4.4 | 20.7 ± 5.7 | **0.002** |
| Predicted peak VO_2_ (%) | 64.1 ± 15.4 | 72.8 ± 21.7 | **< 0.001** |
| VE/VCO_2_ slope | 29.0 ± 5.1 | 27.6 ± 5.4 | **0.044** |
| AT (ml/kg/min) | 11.9 ± 2.6 | 13.4 ± 2.9 | **0.001** |
| Peak WR (watts) | 101.0 ± 38.5 | 120.5 ± 44.5 | **< 0.001** |
| Relative peak WR (watts/kg of body weight) | 1.14 ± 0.36 | 1.35 ± 0.40 | **< 0.001** |
| RQ/RER | 1.2 ± 0.3 | 1.2 ± 0.2 | 0.220 |
| Peak VE (L/min) | 50.2 ± 19.9 | 67.2 ± 22.6 | **< 0.001** |
| WR at AT (watts) | 42.9 ± 20.2 | 68.1 ± 25.0 | **< 0.001** |
| Relative WR at AT (watts/kg of body weight) | 0.49 ± 0.21 | 0.78 ± 0.24 | **< 0.001** |
| **Ultrasound indexes^a^** | | | |
| EF (%) | 30 (28 - 40) | 35 (30 - 45) | **< 0.001** |
| **Abbreviations: CR,** cardiac rehabilitation; **Rest VO_2_,** oxygen uptake at rest; **Peak VO_2_,** peak oxygen uptake; **Predicted peak VO_2_,** predicted peak oxygen uptake; **VE/VCO_2_ slope,** the slope of the ventilatory equivalent for carbon dioxide; **AT,** anaerobic threshold; **Peak WR,** peak work rate; **RQ,** respiratory quotient; **RER,** respiratory exchange ratio; **Peak VE,** peak minute ventilation; **WR at AT**, the workload at the anaerobic threshold; **EF,** ejection fraction; **SD,** standard deviation.  a Values are presented as median (25^th^ - 75^th^ percentiles).  b Values are presented as mean ± SD. | | | |

| **Supplementary Table 2. Differences in absolute values of parameters of interest between exercise training groups after the cardiac rehabilitation program.** | | | |
| --- | --- | --- | --- |
| **Variables of the CR program** | **HIIT Group**  (19 patients) | **COM Group**  (25 patients) | **P value** |
|  | ***Difference*** | ***Difference*** |  |
| **Minnesota Living With Heart Failure Quality of Life Questionnaire^a^** | | | |
| Physical score (units) | - 4 (-9 – 0) | - 3 (-11 – -0.5) | 0.962 |
| Emotional score (units) | - 1 (-6 – 0) | 0 (-2 – 0.5) | 0.557 |
| Total score (units) | - 10 (-20 – 1) | - 7 (-17.5 – 0.5) | 0.704 |
| **1 Repetition Maximum test^a^** | | | |
| Quadriceps (kg) | 6 (4 – 15) | 9 (3.5 – 12) | 0.785 |
| Chest muscles (kg) | 5 (3 – 12) | 13 (5 – 21) | **0.039** |
| **Muscular Endurance^a^** | | | |
| Quadriceps (repetitions) | 5 (2 – 8) | 3 (2 – 5) | 0.294 |
| Chest muscles (repetitions) | 2 (1 – 6) | 6 (3 – 10) | **0.002** |
| **Cardiopulmonary exercise testing indexes^b^** | | | |
| Rest VO_2_ (ml/kg/min) | - 0.54 ± 0.95 | - 0.17 ± 1.31 | 0.312 |
| Peak VO_2_ (ml/kg/min) | 3.16 ± 4.85 | 1.66 ± 4.39 | 0.290 |
| Predicted peak VO_2_ (%) | 11.42 ±15.92 | 6.80 ±14.56 | 0.322 |
| VE/VCO_2_ slope | - 2.04 ± 5.16 | - 0.86 ± 3.71 | 0.380 |
| AT (ml/kg/min) | 0.75 ± 1.85 | 1.99 ± 3.02 | 0.101 |
| Peak WR (watts) | 18.63 ± 17.64 | 20.04 ± 20.38 | 0.811 |
| RQ/RER | - 0.09 ± 0.41 | - 0.03 ± 0.15 | 0.462 |
| Peak VE (L/min) | 16.61 ± 13.29 | 17.39 ± 15.55 | 0.861 |
| WR at AT (watts) | 19.74 ± 10.08 | 29.24 ± 15.20 | **0.023** |
| Peak P_ET_CO_2_ (mmHg) | - 6.08 ± 8.73 | - 1.32 ± 7.68 | 0.062 |
| **Ultrasound indexes^a^** | | | |
| Ejection Fraction (%) | 5 (0 – 5) | 5 (0 – 5.5) | 0.587 |
| **Abbreviations:** **CR,** cardiac rehabilitation; **Rest VO_2_,** oxygen uptake at rest; **Peak VO_2_,** peak oxygen uptake; **Predicted peak VO_2_,** predicted peak oxygen uptake; **VE/VCO_2_ slope,** the slope of the ventilatory equivalent for carbon dioxide ; **AT,** anaerobic threshold; **Peak WR,** peak work rate; **RQ,** respiratory quotient; **RER,** respiratory exchange ratio; **Peak VE,** peak minute ventilation; **WR at AT**, the workload at the anaerobic threshold; **P_ET_CO_2_,** end-tidal partial pressure of CO_2_; **EF,** ejection fraction; **SD,** standard deviation.  a Values are presented as median (25^th^ - 75^th^ percentiles).  b Values are presented as mean ± SD. | | | |
